# Global warming as a detectable thermodynamic marker of Earth-like extrasolar civilizations: the case for a telescope like Colossus

**DOI:** 10.1017/S1473550414000585

**Published:** 2015-03-17

**Authors:** Jeff R. Kuhn, Svetlana V. Berdyugina

**Affiliations:** 1 University of Hawaii, Institute for Astronomy, 34 Ohia Ku St, Pukalani, Maui, HI, 96768, USA; 2 NASA Astrobiology Institute, University of Hawaii, Institute for Astronomy, 2680 Woodlawn Dr, Honolulu, HI 96822, USA; 3 Kiepenheuer Institut fuer Sonnenphysik, Schoeneckstr. 6, 79104 Freiburg, Germany

**Keywords:** civilization biomarker, extraterrestrial civilizations, global warming, Kardashev Type I civilizations

## Abstract

Earth-like civilizations generate heat from the energy that they utilize. The thermal radiation from this heat can be a thermodynamic marker for civilizations. Here we model such planetary radiation on Earth-like planets and propose a strategy for detecting such an alien *unintentional* thermodynamic electromagnetic biomarker. We show that astronomical infrared (IR) *civilization biomarkers* may be detected within an interestingly large cosmic volume using a 70 m-class or larger telescope. In particular, the Colossus telescope with achievable coronagraphic and adaptive optics performance may reveal Earth-like civilizations from visible and IR photometry timeseries’ taken during an exoplanetary orbit period. The detection of an alien heat signature will have far-ranging implications, but even a null result, given 70 m aperture sensitivity, could also have broad social implications.

## Introduction

The detection of extraterrestrial life will be an important scientific achievement. Searches for *intentional* or *beaconed* alien signals have made great advances in sensitivity (Backus & The Project Phoenix Team 2002; Phoenix Project 2002) but, until we detect a signal, these results are difficult to interpret. Unambiguous conclusions from such null results require tenuous assumptions about alien sociology and ‘cosmological principles.’ Furthermore, our imaginations simply may not encompass the alien communication modes we could encounter. Gaining a better understanding of our evolution, either as one of many realizations of some biological ‘Copernican Universalism,’ or as a unique ‘Anthropic requirement’ (cf. Michaud 2007) of physical and biological laws, needs more than just a binary answer to the question ‘are we alone in the universe?’ The last decade of exoplanet studies have taught us that the Earth probably ‘is not special’ on a cosmic scale (e.g., Pepe *et al.* 2011; Kopparapu *et al.* 2014). Could it be that advanced life also ‘is not special’? We seek biomarkers whose completeness can be quantified with respect to the cosmic volume probed versus civilization type or advancement. With this aim, we propose here a strategy to find *unavoidable* thermodynamic alien biomarkers that would be difficult to hide from our remote sensing technologies.

The radio or optical signalling power that ‘leaks’ from an advanced civilization is difficult to predict (e.g., Loeb & Zaldarriaga 2007; Forgan & Nichol 2011), but such power estimates are a useful classification scheme. Kardashev (1964) argued that advanced civilizations could be distinguished by the radio power they can generate. He called the Earth early Type I, Type II civilizations harness the full power of their host star, and a Type III can harness the power of its Galaxy. The idea of classifying a planetary civilization by its power generation was further refined by defining an index *K* = log_10_(*P*)/10–0.6, where *P* is the average power (in Watts) consumed by the planetary civilization (Shklovskii & Sagan 1966). On this scale Earth has *K* = 0.7, since *P* was about 15 TW in the year 2010.

Dyson (1960) suggested searching for the thermodynamic signature of aliens capable of building a star-enclosing ‘biosphere’ at roughly their planet's orbit radius. He argued that they could capture their star's luminosity for their uses while unavoidably (due to the First Law of Thermodynamics) radiating the thermal waste into space with a black-body temperature near the planet's equilibrium temperature. While astronomical infrared (IR) surveys have not turned up any such *Type II* or *III* candidates (Carrigan 2009), the concept of using alien thermal waste is a powerful unintentional ET biomarker.

Waste heat is a nearly unavoidable indicator of biological activity, just as the energy that civilization consumes is eventually reintroduced into the planetary environment as heat. On planetary scales, biologically produced heat tends to be spatially clustered, just as an ET civilizations’ technological heat is difficult to distribute uniformly. Planetary surface topography and the efficient tendency for population to cluster in agrarian and urban domains leads to heat ‘islands’ (cf. Rizwan *et al.* 2008).The temporal and spatial distribution of this heat can be an observable ‘fingerprint’ for remote sensing of civilizations. Here we argue that we may soon be in a position to detect this thermodynamic signal from Type I, nearly Earth-like civilizations.

## Modelling unintentional civilization heat signals

### Power consumption parameterization

We believe that thriving Type I civilizations evolve towards greater power consumption. Notably, there is a strong correlation between our power consumption and society's accumulated information content. Humanity collects information with a doubling time of about 3 years (Gantz *et al.* 2008), and our power consumption is increasing faster than the population (IEA 2013). Even an advanced efficient civilization will have vast power requirements because of the fundamental information-theoretic energy cost to acquire and manipulate its knowledge base (cf. Maruyama *et al.* 2009).

It is useful to compare civilization's energy flux with its host star's flux. We define a quantity

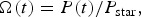

where *P*_star_ is the stellar power intercepted by the planet and *P*(*t*) is the civilization's power consumption. This dimensionless number is a possible ‘advancement’ metric. The Earth currently has Ω_E_ = 0.0004 (IEA 2013) and 2000 years earlier had Ω = 10^−7^ (Malanima 2011). In comparison, the present optical light power generation is a tenth of our power production, while human biological heat production corresponds to about Ω = 3 × 10^−5^ (calculated from the population number). The power associated with terrestrial plant photosynthesis, which fuels most terrestrial biology, dominates all of these sources and is Ω_ph_ = 0.002 (Nealson & Conrad 1999). It is interesting to note that human technology already generates nearly 20% of the Earth's biological energy production (this is the ratio of Ω_E_ to Ω_ph_). Note that the photosynthetic energy consumption which is stored (for example, as oil or coal) eventually fuels much of our net terrestrial heat production. We expect advanced civilizations to eventually surpass their planetary biological energy production.

Planetary temperatures are determined by balancing the energy inputs (stellar, planetary heat sources, etc.) with the thermal energy radiated from the planet. Since most of the consumed power is eventually returned to the planet environment as heat, a civilization with growing power needs will eventually reach a point where they become uncomfortably warm. Perhaps a sufficiently advanced civilization can engineer the planet's albedo and radiative efficiency to moderate such global warming or they might find an energetically favourable way to radiate this heat away from the planet above the atmosphere, but this must also be detectable remotely. However, it is likely that such measures can help only temporarily if *P* continues to grow. Finding a planet that is ‘too hot’ compared to its stellar heat budget with a geographic temperature excess that is not geothermal might also be a sign of advanced life. We assume that eventually mass-migration to another planet can become energetically advantageous. Thus, for somewhat general reasons we may expect planetary civilizations to evolve towards a maximum Ω(*t*) = Ω_0_ ≤ 1 and then either moderate their power consumption or undergo planetary migration. The value of Ω_0_ is uncertain.

Exoplanetary geography and the alien technology and heat tolerance will determine Ω_0_. Since we have already achieved Ω_E_ = 0.0004, it is likely that more advanced alien civilizations can live with Ω_0_ values larger than Ω_E_ and perhaps close to 1. The condition Ω_0_ = 1 defines a natural transition point for Type I civilizations to become interplanetary (or even interstellar) colonists. Theories of space colonization were considered earlier but for a different reason (e.g., O'Neill 2000). The most advanced civilizations could engineer massive starlight power sources rather than fission, fusion or fossil energy from planetary resources. This could be remotely observable as a planetary scale albedo reduction. If all of the incident stellar power is used for useful work and the waste heat returned to the planet such a civilization could have Ω ≈ 1. In general, the temperature of the waste heat depends on the alien technology, but we expect it to be close to the planet's radiative temperature in order to achieve the highest Carnot efficiency. This is the case with terrestrial urban heat islands that are up to 10 °C warmer than the surrounding environment (e.g., Kim 1992, Rizwan *et al.* 2008). Thus, we model here an advanced Earth-like Type I civilization (Ω_E_ < Ω < 1), which radiates heat at nearly the same temperature as the planetary environment.

### Model assumptions and simulations

In a simple but instructive formulation, we describe here the problem in terms of timeseries constructed from the Earth's simulated visible and IR photometric variability as they could be seen by a distant observer. From the outset we note that there are many ways that any detection strategy can be confused (e.g., by perpetual clouds, peculiar ocean/land geography, etc.). Therefore, this algorithm is an example technique that illustrates how multi-wavelength photometric variability data may be used to infer life and civilization biomarkers.

**Fig. 1.**
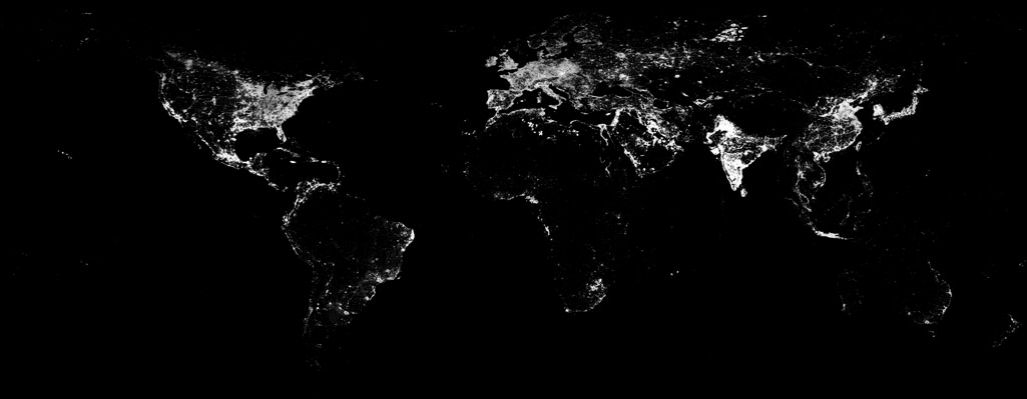
Man-made visible light on the Earth in 2011. From DMPS/NASA. The brightest pixels in this 0.5 × 0.5 degree resolution map have a radiance of about 0.05 × 10^−6^ W/cm^2^/sr/micron.

**Fig. 2.**
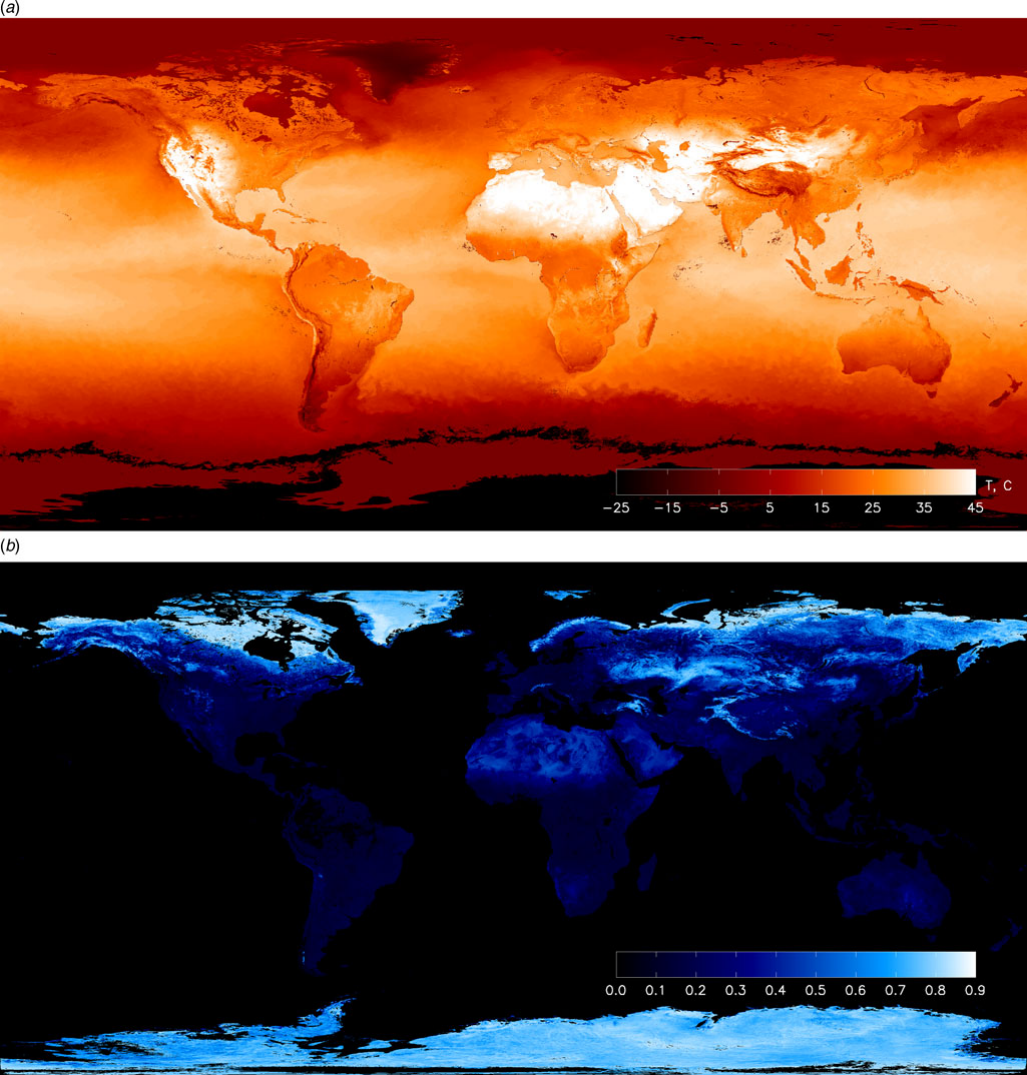
The global temperature (top) and albedo (bottom) distribution of Earth used for modelling an Earth-like natural variability in the visible and infrared. From NEO/NASA. (http://neo.sci.gsfc.nasa.gov).

**Fig. 3.**
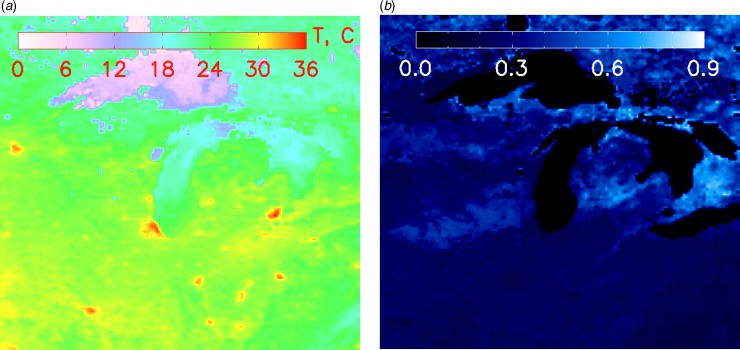
Expanded view of a representative North American region illustrating temperature perturbation due to cities (left, heated cities are seen in red) and corresponding surface albedo (right). From NEO/NASA.

**Fig. 4.**
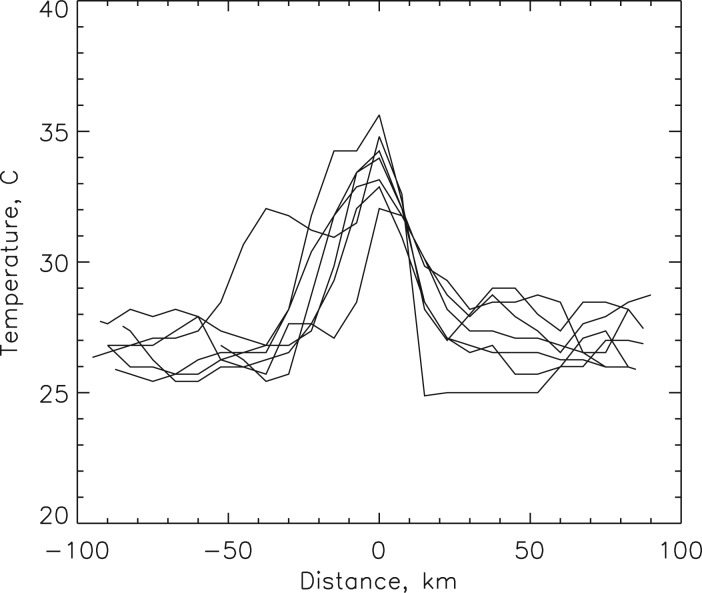
Temperature profiles of some urban heat islands seen in Fig. 3. Pixels in the original data were approximately 11 km.

The Earth's temperature, albedo and visible emissivity (due to man-made lights) are accurately known (NASA 2014). For example, Fig. 1 shows the night-time visible brightness from the year 2011. Similarly the effective blackbody temperature and albedo of the Earth's surface are illustrated in Fig. 2. It is easy to see the thermal signature of cities in Fig. 3. Here we see that Midwestern cities near Michigan appear with a peak temperature excess as large as 10 °C compared to their surroundings during daytime summer conditions (Fig. 4). In contrast, the terrestrial albedo of this region has a relatively weak geographic variability, except for the land–water variation (Fig. 3). This observation can be used to isolate a civilization thermal biomarker from the effects of planetary albedo variability. Figure 4 shows samples of the temperature variation along east–west cuts through several heat islands visible in Fig. 3.

**Fig. 5.**
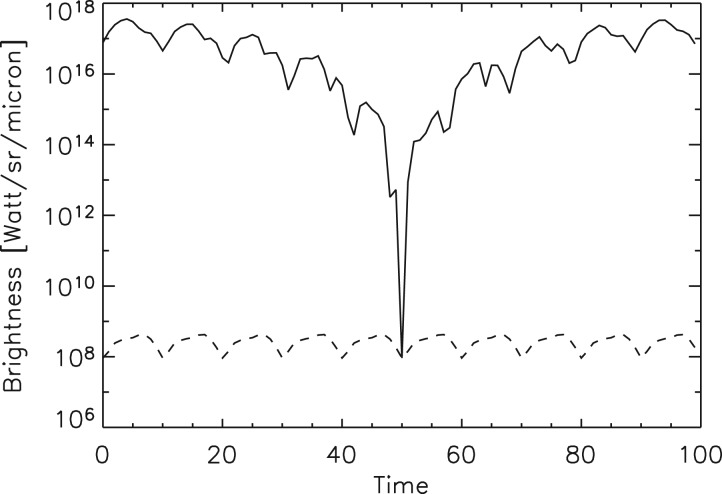
The spatially integrated visible brightness variation of a simulated Earth: solid line is due to surface reflectance of the sunlight using albedo from Fig. 2, and dashed line is due to the man-made light signal using data from Fig. 1 (if it could be isolated from albedo and Earth-scattered sunlight). Here the Earth-like planet is assumed to rotate with the 10-day period and revolve around the star with the 100-day period. Horizontal time axis units here are simulated days.

**Fig. 6.**
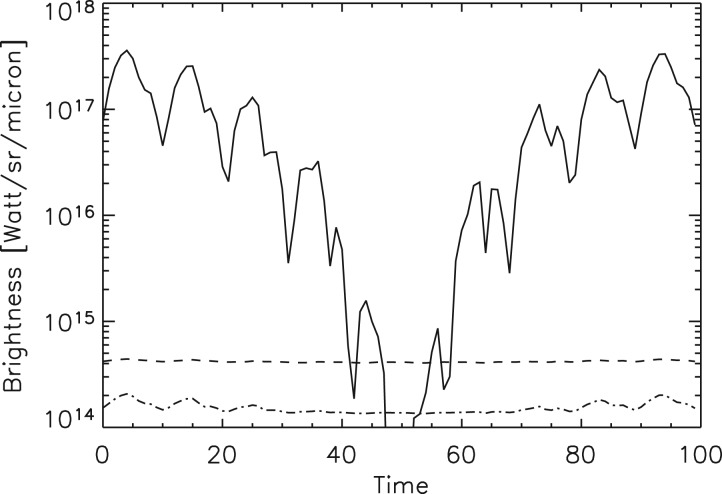
The Earth's natural brightness variability in the infrared is compared to the visible brightness as seen by a distant observer for the same planet as in Fig. 5. The visible brightness variability is plotted with a solid line (same as in Fig. 5), infrared brightness at 5 and 10 μm are shown with dashed and dash-dotted lines, respectively. Note that the infrared rotational modulation is larger at 10 μm than at 5 μm. Horizontal time axis units are simulated days.

We use the NASA Earth Observations (NEO) terrestrial data for surface temperature and albedo shown in Fig. 2 in order to simulate planet natural variability. We integrate the reflected Solar illumination and planet's visible flux due to sunlight reflectance computed from the albedo map. Thermal radiation at 5 and 10 μm is integrated using the temperature map. These maps are available with 0.1° × 0.1° resolution, and were integrated using this grid. The integration was carried out over the projected planet disc as seen by a distant observer. In order to obtain light curves, we calculate fluxes at a number of orbital phases for arbitrary planetary stellar orbit geometry. For simplicity, we assume Lambert surface scattering and describe the brightness in spectral irradiance units (W ster^−1^ μm^−1^). The calculated visible brightness variability is shown in Fig. 5 with solid line, and the IR flux light curves in Fig. 6 with dashed and dash-dotted lines. Analogously, we integrate man-made light flux from the map shown in Fig. 1. The light curve is shown in Fig. 5 with dashed line. Our simulated timeseries depend on three parameters *i*, θ and *T*, where *i* is the orbit inclination to the observer (*i* = 90° is edge on), θ is the inclination of the planet rotation axis to the orbital plane normal direction, and *T* is the planet rotation period in units of the orbit period. ‘Pathological’ geometries with *i* = 0° and θ = 0°, shows no useful photometric variability, but in general other combinations of parameters yield observable biomarkers. We are developing a general photometric inversion code for arbitrary conditions (Berdyugina & Kuhn, in preparation) when 0 < *i* < 90 that can reveal the two-dimensional (2D) exoplanetary albedo and temperature but here we illustrate results for the simplest case of a civilization biomarker signal from an approximately edge-on exoplanet orbit. As long as our observational timeseries has short-enough sample intervals to capture planetary rotational phase angle increments that resolve the civilization thermal variability our results are not sensitive to the rotation or orbit periods. Thus, for our simulated Earth-like light curves, we take *T* = 0.1 with simulated observations that sample 10 points in each planet rotation of 10 days and planetary orbit period of 100 days.

At short wavelengths the visible brightness of the Earth is completely dominated by scattered sunlight. Figure 5 shows the visible planetary light curve and the visible man-made light sources – four orders of magnitude below the already faint (compared to the nearby star) natural terrestrial rotation and orbit brightness variations. Note that in the visible the albedo causes a large rotational modulation, which vanishes when the planet dark-side faces the observer (near day 50). For orbit inclinations farther from 90° the orbital modulation is smaller.

At wavelengths longer than about 3 μm the geographic albedo modulation is small as the trend in Fig. 6 shows for simulated 5 and 10 μm observations. The rotational IR variability illustrated in these curves is due primarily to the natural land–water geography. A 2D inversion, as is done for detecting stellar sunspots in spatially unresolved timeseries photometry (Berdyugina *et al.* 2002) can reveal the planetary landmass distribution information in both latitude and longitude if 0 < θ < 90 (Berdyugina & Kuhn, in preparation). Note that the mean planetary flux at 10 μm is larger than at 5 μm because of the planetary mean temperature, and the relative planetary reflected flux variations in the IR are significantly smaller than at shorter wavelengths.

Short-wavelength photometry is primarily sensitive to the planet's albedo, so a straightforward technique to separate the thermal signal from natural reflected light planetary variability is to scale and subtract the short-wavelength temporal variability signal from the 10 μm IR flux variation. When we do this with the simulated Earth-data, we find a residual variability of about 0.8% at 5 μm and a variation of 0.5% at 10 μm. Currently the Earth's man-made terrestrial heat signal is less than this, so this simple technique is sensitive to more advanced civilizations with Ω > 0.01 approximately.

**Fig. 7.**
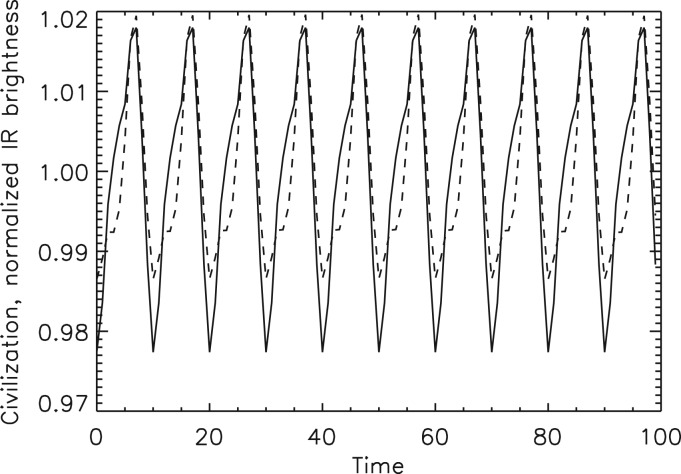
A simulated Ω ≈ 0.01 civilization signal at 10 μm (solid line) based on Earth's man-made visible light geographic distribution (Fig. 1) scaled to represent civilization heat (as described in the text). This signal was combined with Earth's natural geographic variability (Figs. 2) and extracted using our simple algorithm described in the text. The inferred civilization thermal signature is overplotted with dashed line. The rotational phase and amplitude of the exocivilization signal are reasonably recovered. Horizontal time units are simulated days.

To illustrate this biomarker we simulate an exo-civilization by scaling the man-made visible light brightness map shown in Fig. 1 to produce a civilization thermal signal that is about 50 times larger than our current technological heat production. We use this to describe the IR emissivity of our hypothetical Ω≈0.01 advanced civilization. The thermal flux variations at 10 μm due to such a civilization are shown in Fig. 7 with solid line. This civilization temperature map is added to the natural terrestrial temperature variations from which we compute the visible, short- and long-wavelength IR photometry as we did before using Fig. 2. Then, following our algorithm, we scale and subtract the short-wavelength IR temporal variability signal from the 10 μm IR flux variation. This is done by linearly regressing the short-wavelength variability against the longer-wavelength flux variation and using the residual of this to represent non-albedo flux variations. The dashed line curve in Fig. 7 shows the civilization thermal variability light curve recovered with this procedure. A comparison with the overplotted original civilization thermal signal (in the absence of natural terrestrial variability) demonstrates that both the magnitude and the rotational phase of the civilization biomarker can be determined with this basic regression technique.

### Perturbations on the thermal civilization signal

Our simple model illustrates the form of the planetary signals and demonstrates one algorithm for extracting a thermal civilization signal. Of course there are extrasolar planets where this model is incomplete (for example a planet covered by clouds, e.g., Gómez-Leal *et al.* 2012, or covered by water) and this detection scheme would fail. On the other hand, a cloud-shrouded planet will show a distinct thermal signature and could be eliminated from a cosmic census. Detailed atmospheric spectroscopy of exoplanetary candidates is also possible with the same detector system that searches for thermal biomarkers so that a combination of thermal and spectroscopic observations could be used to further expand our remote sensing tools for exoplanetary life.

These timeseries light curves illustrate some other important characteristics that the data-processing algorithms may use to identify potential false-positive civilization biomarkers (like geophysical contributions):

• An orbital modulation is due to the dark/light side of the planet as it becomes visible to the Earth that may reveal transient planetary heating information.
• The higher frequency modulation shows the effect of planetary surface features rotating across the visible hemisphere as characterized primarily by the albedo.
• The visible light flux and star-heated thermal flux are anti-phased at high (rotation) frequencies, because the scattered light is largest when the albedo is highest and the absorbed energy is less.
• A civilization signal may be anti-correlated on orbital times because the night-time civilization power dissipation may be greater and detectible.
• Much hotter thermal variability using multiple IR wavelengths is a likely marker of geothermal sources.

Our simple algorithm can detect a simulated Ω > 0.01 terrestrial-like heat-signal. Isolating this from geothermal variability might be done in general by measuring the temperature versus phase of the thermal signal. For instance, isolated ‘hot’ contributions could be further studied for evidence of a geothermal origin for other gas spectroscopy information. We anticipate circumstances when other observable constraints on the natural variability can play a supplemental role for civilization detection.

Our approach here illustrates that without prior knowledge of the planet's albedo and heat distributions it is possible *in principle* to extract an alien civilization signature from sensitive visible and IR flux measurements, *even when natural stellar heating dominates the total planetary flux*. By constraining the planet's geographic albedo with other astronomical observations (e.g., spectroscopy or 2D inversion) the robustness of the solution could be further improved, subject to other observational constraints or *a priori* assumptions. We now argue that an interesting net scattered visible and thermal flux from Earth-size or larger planets within 60 light-years can be detectible with a large telescope and sensitive coronagraph.

## Detection strategy

### Terrestrial HZ planets in the Solar neighbourhood

While our primary goal is to *detect* advanced alien civilizations, we believe the incentive to communicate with neighbours, if they are found, will be irresistible. Consequently we have great interest in finding life which is within communication light-travel times of Earth, say 20 pc, and life which has a chemical basis similar to humanity's that makes intelligible communication a more likely possibility (see also arguments by Kasting *et al.* 2014). Therefore, here we focus our estimates on water-dependent biology and, for simplicity, consider terrestrial exoplanets in the habitable zone (HZ) around their host stars defined for liquid water conditions. We have used a simplistic (and conservative) temperature-based definition of HZ, which effectively assumes an Earth-like atmosphere, Kasting *et al*. (2014) describe how water on HZ planets may be more generally characterized.

It is known that A, F, G, K and M main-sequence stars live longer than a few 100 Myr, and may be old enough to spawn advanced civilizations, or have sufficient lifetimes to justify colonizing their planets. There are about 650 such stars brighter than V-magnitude 13 within 20 pc of the Sun (SIMBAD), and at least 30–50% of them should have terrestrial planets (Pepe *et al.* 2011; Kopparapu *et al.* 2014). The HZ distance, *d*_HZ_, for Earth twins orbiting main-sequence stars is scaled by the stellar effective temperature (i.e., flux) and varies between 0.07 AU (for a 3000 K M star) up to 10 AU at A-type star temperatures of 11 000 K.

**Fig. 8.**
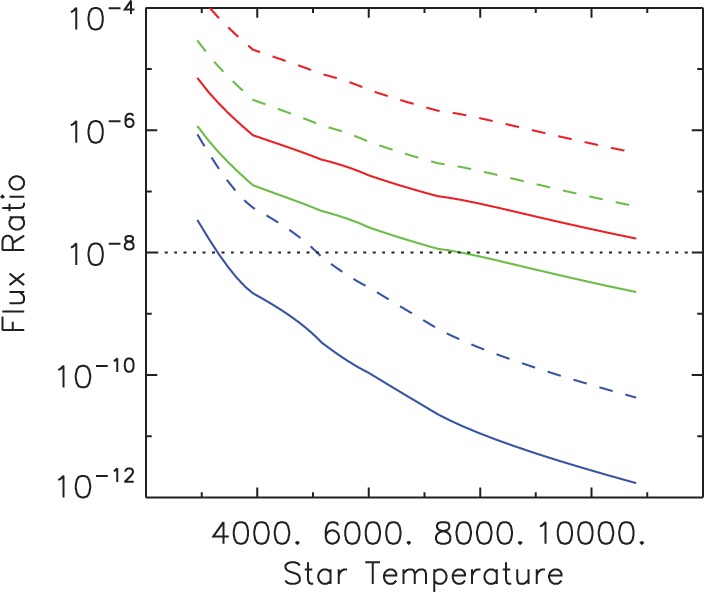
Flux contrast for a planet in the HZ versus star temperature in scattered stellar light (blue), in planet emission at the wavelength of 5 μm (green) and in emission at 10 μm (red). Solid lines show contrast of Earth-radius planets and dashed lines correspond to five Earth-radius planets. The Earth-like geometrical albedo 0.3 was assumed.

We argued in the Section ‘Modelling unintentional civilization heat signals’ that finding unintentional extraterrestrial civilization (ETC) heat footprints requires sensitive timeseries in both the visible and IR over at least an orbital period. It is necessary therefore to estimate the optical and IR contrast of the planet with respect to the direct stellar light. The relative *optical* flux of the light scattered from an habitable zone Earth (HZE) compared to the direct stellar flux depends only on the star temperature, the planet radius, *R*, and albedo, *A*. It equals *AR*^2^/4*d*_HZ_^2^ and is plotted in Fig. 8 (blue curves). The increase of the reflective contrast towards cooler stars is promising, but for an HZE around the Sun it is 10^−10^, and no telescope has yet achieved this contrast sensitivity to detect optical light from such planets. The *thermal* emission from HZEs has higher contrast because the stellar IR flux is relatively fainter than the cooler planet emission (Fig. 8, green and red curves). It is at wavelengths between 5 and 10 μm that we have the greatest sensitivity for detecting ETC waste heat flux. We conclude that many terrestrial HZ planets are potentially detectable if we can achieve IR contrast sensitivity of at least 0.5 × 10^−7^ or visible reflected light contrast sensitivity of about five times better. Several interesting candidates have been already found in the Solar neighbourhood (see Table 1).

**Table 1.** A sample of known super-Earth planets within their circumstellar habitable zones in the Solar neighbourhood (d < *20 pc*)

Planet	*M*/*M*_⊕_	*R*/*R*_⊕_	Stellar Sp.	*d* (pc)	Ang. sep. (mas)	*V* (mag)	Ref
Gliese 163 c	>7.2	–	M3.5V	15.0	8.4	11.8	[6]
HD40307g	>7.1	–	K2.5V	12.8	47	7.2	[7]
HD85512 b	>3.5	–	K5V	11.2	23	7.7	[2]
Gliese 667C c	>4.3	–	M1.5V	6.8	18	10.2	[3–5]
Gliese 832 c	>5.0	–	M1.5V	4.9	33	8.7	[1]
Kapteyn b	>4.8–	–	dM1	3.9	43	8.8	[8]
α Cen A HZ	–	–	G2V	1.3	944	0.0	[9]
α Cen B HZ	–	–	K1V	1.3	558	1.3	[9]
α Cen C HZ		–	dM5e	1.3	38	11.1	[9]

References: [1] Wittenmyer *et al.* (2014); [2] Pepe *et al.* (2011); [3] Delfosse *et al.* (2013); [4] Anglada-Escude *et al*. (2012); [5] Feroz & Hobson (2014); [6] Bonfils *et al.* (2013); [7] Tuomi *et al.* (2012); [8] Anglada-Escude *et al*. (2014); [9] HZ angular sizes of the α Cen components as the nearest stars to the Sun are listed for comparison.

### Next-generation large telescope requirements

The near-future large optical and IR telescopes will be barely able to spatially resolve HZE from their stars. Therefore, we focus here on rather practical and affordable next-generation telescopes for observations of exoplanets as point-like sources which are resolved from their stars and, therefore, can be investigated with indirect inference techniques similar to the one proposed in the Section ‘Modelling unintentional civilization heat signals’ (but see Schneider *et al.* 2010 for a far future perspective).

The number of possibly observable HZ planets as resolved light sources increases rapidly with telescope diameter, *D*, because the telescope resolution improves proportional to *D*, so that the limiting stellar distance to resolve an HZ exoplanet must also increase proportional to *D*. Thus, the cosmic volume sampled with sufficient angular resolution to measure the exoplanet light scales as *D*^3^. It follows that a large telescope can have enormously larger detection sensitivity. The telescope must satisfy three stringent optical requirements, which are discussed in this section:

1) high level of scattered light suppression in order to see the faint terrestrial planet against the optical ‘glare’ of the nearby star;
2) sufficient sensitivity for detecting enough photons from the planet to allow statistical analysis of its variability;
3) low-enough thermal emissivity so that the planetary IR flux is not lost in the terrestrial thermal background.

The first requirement depends on excellent adaptive optics performance and coronagraphic scattered light suppression. The second relies on having a large telescope aperture, and the third goal is attained with careful control of the primary mirror and secondary optics emissivity.

Glare from the central star is due in part to limitations in the telescope adaptive optics that do not completely correct the atmosphere-scattered light. Additional scattered-light is caused by diffraction from the telescope. Suppressing this background requires both a coronagraph and adaptive optic systems. Instruments for ground-based extrasolar planet detection are currently being built to yield contrast sensitivity of 10^−8^ at angles larger than 6λ/*D* on 8 m telescopes, i.e., angles larger than 0.12 arcsec at λ = 800 nm, (*D* is the telescope diameter; Macintosh *et al.* 2007; Roelfsema *et al.* 2011). It is possible that higher contrast will be achieved from space and with more advanced coronagraphs (Guyon *et al.* 2006), but 10^−8^ at 6λ/*D* is a practical goal for future ground telescopes.

**Fig. 9.**
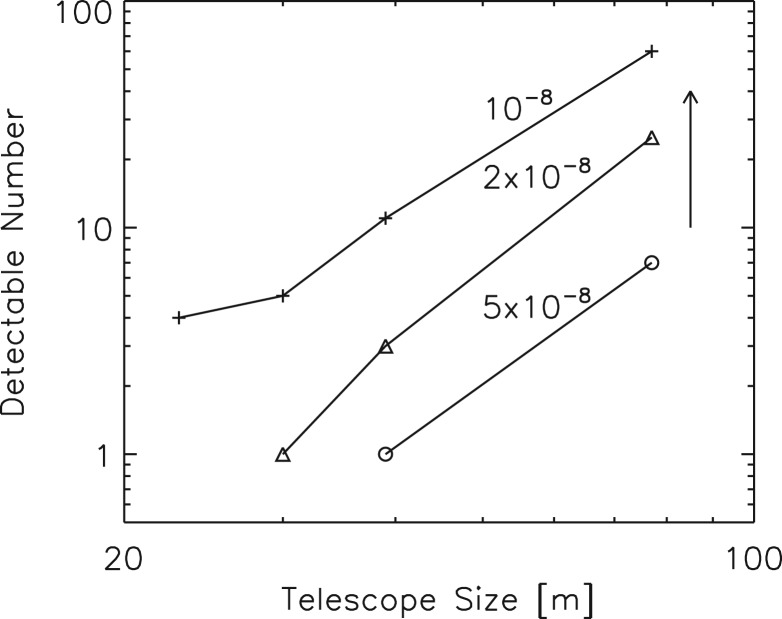
Maximum number of detectable Earth-size HZ planets (assuming 1 per star and Ω≈1) versus telescope size. ‘Star’ symbols show number detectable at 5 μm due to thermal emission assuming 5 × 10^−8^ contrast at an angle of 2λ/*D* from the host star and at 500 nm with five times smaller contrast at 20λ/*D*. ‘Diamond’ symbols show detectable number with corresponding IR contrast at 2 × 10^−8^ and ‘plus’ symbols show number at 10^−8^. Up arrow shows the increase in detection numbers if HZ planets have a radius twice the Earth's.

The advantage of a larger telescope for detecting ETCs is overwhelming. Figure 9 shows that simultaneous visible (0.5 μm) and IR (5 μm) measurements of HZEs are possible for a number of stars with a telescope larger than about 70 m. There are possibly ~30 nearby HZ bright systems detectable with a 75 m telescope with intermediate contrast having a median distance of 5.9 pc and V magnitude of 8.6. A sample of currently known terrestrial planets in the ‘restricted’ HZ (Table 1) within 20 pc will be resolved by such a telescope. On the contrary, the currently planned ‘World's Largest Telescopes’ (WLTs) with apertures of <39 m (TMT 30 m, E-ELT 39 m, and GMT 24 m) will not reach a significant number of ETC candidates. None of the known HZ super-Earths could be resolved from their central stars by them. Space telescopes have no overwhelming advantage for this detection problem, since it depends so strongly on aperture (however, there is some advantage for IR measurements because of cold telescope optics in space).

### The Colossus telescope

A promising and practical WLT-concept with *D* = 74 m is the Colossus telescope (Colossus 2012; Kuhn *et al*. 2014; Moretto *et al.* 2014). It is a scalable system of 8 m off-axis telescopes to create a large, nearly filled-aperture interferometer-style instrument. It was demonstrated that off-axis telescopes have great advantages in terms of emissivity, diffraction-limited energy concentration, and higher dynamic range (see review by Moretto & Kuhn 2014). In particular, the coronagraphic performance of off-axis telescopes is exceptionally good for faint companion detections (Kuhn & Hawley 1999). A precursor of the Colossus was a High Dynamic Range Telescope proposed by Kuhn *et al.* (2001), which served also as a basis for the GMT. In contrast to other WLTs, the Colossus telescope is optimized for high-contrast imaging of a narrow field around a bright source, i.e., it is especially suitable for direct measurements of exoplanets, which is a driving science case for this project. Using larger and relatively few mirror elements dramatically reduce the amount of the scattered light. The Colossus telescope at a mountain site could collect about 10^3^ photons s^−1^ in the 4.5–5.5 μm band from a terrestrial planet at a distance of 5 pc. With realistic telescope emissivity of 0.05 (e.g., similar to Gemini), a 3 h HZE observation at wavelengths near 5 μm could measure an Ω≈0.05 civilization thermal flux. Nearer stars, larger-than-Earth planets (Fig. 8), or hotter ETC heat-dumps will be more sensitively measured. Thus several nights of observations with the Colossus telescope distributed over the planet's orbital and rotation periods could detect ETCs with Ω > 0.05 (Section ‘Modelling unintentional civilization heat signals’). Such telescopes will also be capable of detecting terrestrial-like biological life using spectroscopic biosignatures (e.g., Berdyugina *et al.* 2014).

In the visible, the larger star-HZ separation angle (in units of λ/*D*) and possibility to observe a planet's polarized scattered light to further suppress starlight (e.g., Berdyugina *et al.* 2011) should allow even better contrast sensitivity. In this case, it will be possible to obtain the planet's rotationally modulated albedo and thermal signatures from many ETC candidates.

## Discussion

We parameterized Kardashev Type I ETCs with a dimensionless quantity, Ω, and considered their detection based on their unintentional waste heat. Our approach is to look for civilization's geographically clumpy thermal excess by analysing the time dependence of a planet's thermal radiation and reflected light. A low-temperature (≥300 K) general excess power could also be a civilization marker if it is distinguishable from the background thermal radiation of the planet. As civilizations are pushed towards utilizing photonic stellar power to avoid global warming, planets with unusually low albedo can be prime advanced civilization candidates. A 2D inversion of the visible light-curves of exoplanets could indicate regions of unusually low albedo that may also point to evidence of advanced life.

The IR flux from the Earth-facing exoplanet hemisphere will vary due to the planet's rotation and its orbital motion but, in general, its time variation will be distinct from the natural planetary albedo effects. We have illustrated this with a simple model based on the terrestrial albedo and man-made light-signal distributions. A multi-wavelength analysis shows that the longitudinal distribution of planetary waste heat can be distinguishable from time variations caused by longitudinal variation in the planet's albedo. We found that alien heat sources with more than 1% of the stellar illumination power (Ω ≥ 0.01) may be identified with our approach. In principle, planets with large natural geothermal sources could also be distinguished thermally or spectroscopically because of their higher radiative temperature. Pervasive clouds or alien power distribution networks that mimic natural stellar planetary heat would make this biomarker difficult to detect and should be ruled out of a statistical census sample.

The technology of civilizations only slightly more advanced than the Earth's may be difficult to distinguish from a planetary biological waste heat signature since these signals may be of comparable amplitude. A civilization might not always be measured by the mechanical or electronic energy it consumes. An interesting example is social insects which by some measures could be considered ‘intelligent’ but whose energy footprint is dominated by biological heat (cf. Korb 2003; Gould & Gould 2007).

We have argued that a large telescope operating from the ground with a powerful adaptive optics system and coronagraph can measure interesting visible and IR flux levels from terrestrial planets around a significant sample of nearby stars, including already known HZ super-Earths. Most of these stars within 20 pc are likely to be older than the Sun, and most of them will have at least one planet. Currently planned WLTs appear to be out of reach of the performance described here. A 70 m class optical system will require new thinking about how to manufacture and control large, precise optical structures. A promising approach is to use what could be described as a scalable system of 8 m off-axis telescopes to create a nearly filled-aperture interferometer such as the Colossus telescope (Kuhn *et al.* 2014).

Regardless of the details of the next WLT, we show that thermodynamic signals of moderately advanced Earth-like civilizations are in principle detectible with current technologies. We believe that it is possible to achieve a *quantifiably complete* neighbourhood cosmic survey for Type I ETC (Kuhn *et al.* 2013) that are within 6 pc of the Sun over a period of about 2 years. Such a program could tell us just how fragile advanced life is, i.e., statistically, how likely it is for an Earth-like civilization to survive. As mentioned earlier, current planet statistics suggests that out of 650 stars within 20 pc at least one quarter would have HZEs. Assuming that one quarter of those will develop Ω ≥ 0.01 civilizations, we arrive at the number of detectable civilizations in the Solar neighbourhood *N*_D_ = 40*f*_s_, where *f*_s_ is the fraction of survived civilizations (i.e., civilizations that form and survive). Hence, even if only one in 20 advanced civilizations survive (including us at the time of survey), we should get a detection. Taking into account the thermodynamic nature of our biomarker, this detection is largely independent of the sociology of detectable ETCs. If we detect none in our neighbourhood, it would lead us to the important conclusion that the survival probability of any given ETC is less than a few per cent after it reaches a certain technological level.

## References

[ref1] Anglada-Escude G., Arriagada P., Vogt S., Rivera E., Butler P., Crane J.D., Shectman S.A., Thompson I.B., Minniti D., Haghighipour N. (2012). A planetary system around the nearby M dwarf GJ 667C with at least one super-Earth in its habitable zone. Astrophys. J. Lett. 751, L16.

[ref2] Anglada-Escude G., Arriagada P., Tuomi M., Zechmeister M., Jenkins J.S., Ofir A., Dreizler S., Gerlach E., Marvin C.J., Reiners A. (2014). Two planets around Kapteyn's star: a cold and a temperate super-Earth orbiting the nearest halo red-dwarf. Mon. Not. R. Astron. Soc. 443, L89–L93.

[ref3] Backus P.R. & The Project Phoenix Team (2002). Project Phoenix SETI Observations from 1200 to 1750 MHz with the upgraded Arecibo telescope. In *Single-Dish Radio Astronomy: Techniques and Applications*, edited by S. Stanimirovic, D. Altschuler, P. Goldsmith, and C. Salter. ASP Conf. Series 278, pp. 525–527.

[ref5] Berdyugina S.V., Pelt J. & Tuominen I. (2002). Magnetic activity in the young solar analog LQ Hya. I. Active longitudes and cycles. Astron. Astrophys. 394, 505–515.

[ref6] Berdyugina S.V., Berdyugin A.V., Fluri D.M. & Piirola V. (2011). Polarized reflected light from the exoplanet HD189733b: first multi-color observations and confirmation of detection. Astrophys. J. Lett. 728, L6 (5 pp).

[ref7] Berdyugina S.V., Kuhn J.R., Harrington D.M., Šantl-Temkiv T. & Messersmith E.J. (2014). Remote sensing of life: polarimetric signatures of photosynthetic pigments as sensitive biomarkers. Int. J. Astrobio., this volume.

[ref8] Bonfils X., Lo Curto G., Correia A.C.M., Laskar J., Udry S., Delfosse X., Forveille T., Astudillo-Defru N., Benz W., Bouchy F. (2013). The HARPS search for southern extra-solar planets. XXXIV. A planet system around the nearby M dwarf GJ 163, with a super Earth possibly in the habitable zone. Astron. Astrophys. 556, A110.

[ref9] Carrigan R.A. Jr. (2009). IRAS-based whole-sky upper-limit on Dyson Spheres. Astrophys. J. 698, 2075–2086.

[ref10] Colossus (2012). A 74 m filled aperture interferometric telescope. http://www.the-colossus.com

[ref12] Delfosse X., Bonfils X., Forveille Th., Udry S., Mayor M., Bouchy F., Gillon M., Lovis C., Neves V., Pepe F. (2013). The HARPS search for southern extra-solar planets XXXIII. Super-Earths around the M-dwarf neighbors Gl433 and Gl667C. Astron. Astrophys., 553, id.A8, 15 pp.

[ref14] Dyson F. (1960). Search for artificial sources of infrared radiation. Science 131, 1667–1668. 17780673 10.1126/science.131.3414.1667

[ref15] E-ELT: European Extremely Large Telescope. http://www.eso.org/sci/facilities/eelt/

[ref16] Feroz F. & Hobson M.P. (2014). Bayesian analysis of radial velocity data of GJ667C with correlated noise: evidence for only two planets. Mon. Not. R. Astron. Soc., 437, 3540–3549.

[ref17] Forgan D.H. & Nichol R.C. (2011). A failure of serendipity: the Square Kilometre Array will struggle to eavesdrop on human-like extraterrestrial intelligence. Int. J. Astrobiol. 10, 77–81.

[ref19] Gantz J.F., Chute C., Manfrediz A., Minton S., Reinsel D., Schlichting W. & Toncheva A. (2008). The diverse and exploding digital universe. IDC White Paper. http://www.emc.com/collateral/analyst-reports/diverse-exploding-digital-universe.pdf

[ref20] Gemini: Gemini South's Secondary Mirror Sports Shiny Silver Coat. http://www.gemini.edu/node/103

[ref21] GMT: Giant Magellan Telescope. http://www.gmto.org/

[ref22] Gómez-Leal I., Pallé E. & Selsis F. (2012). Photometric variability of the disk-integrated thermal emission of the Earth. Astrophys. J. 752, 28, 11 pp.

[ref23] Gould J.L. & Gould C.G. (2007). Animal Architects: Building and the Evolution of Intelligence. Basic Books, New York, 324 p.

[ref24] Guyon O., Pluzhnik E.A., Kuchner M.J., Collins B. & Ridgway S.T. (2006). Theoretical limits on extrasolar terrestrial planet detection with coronagraphs. Astrophys. J. Suppl. 167, 81–99.

[ref25] IEA (2013). International Energy Agency. Key World Energy Statistics. http://www.iea.org/publications/freepublications/publication/name-31287-en.html2

[ref26] Kardashev N.S. (1964). Transmission of information by extraterrestrial civilizations. Sov. Astron. 8, 217–221.

[ref27] Kasting J.F., Kopparapu R., Ramirez R.M. & Harman C. (2014). Remote life detection criteria, habitable zone boundaries, and the frequency of earthlike planets around M and late-K stars. Proc. Natl. Acad. Soc. U.S.A., 111, 12641–12646. 10.1073/pnas.1309107110 PMC4156685 24277805

[ref28] Kim H.H. (1992). Urban heat island. Int. J. Remote Sens. 13, 2319–2336.

[ref29] Kopparapu R.K., Ramirez R.M., SchottelKotte J., Kasting J.F., Domagal-Goldman S. & Eymet V. (2014). Habitable zones around main-sequence stars: dependence on planetary mass. Astrophys. J. Lett. 787, L29, 6 pp.

[ref30] Korb J. (2003). Thermoregulation and ventilation of termite mounds. Naturwissenschaften 90, 212–219. 12743703 10.1007/s00114-002-0401-4

[ref31] Kuhn J.R. & Hawley S.L. (1999). Some astronomical performance advantages of off-axis telescopes. Publ. Astron. Soc. Pacific 111, 601–620.

[ref32] Kuhn J.R., Moretto G., Racine R., Roddier F. & Coulter R. (2001). Concepts for a large-aperture, high dynamic range telescope. Publ. Astron. Soc. Pacific 113(790), 1486–1510.

[ref33] Kuhn J.R., Berdyugina S.V., Harlingten C. & Halliday D. (2013). Finding ETs with infrared light. Astronomy 6, 31–35.

[ref34] Kuhn J.R., Berdyugina S.V., Langlois M., Moretto G., Thiebaut E., Harlingten C. & Halliday D. (2014). Looking beyond 30 meter class telescopes: the Colossus project. In *Astronomical Telescopes and Instrumentation*, Proc. SPIE Ground-based and Airborne Telescopes V, 9145, 91451G.

[ref35] Loeb A. & Zaldarriaga M. (2007). Eavesdropping on radio broadcasts from galactic civilizations with upcoming observatories for redshifted 21 cm radiation. J. Cosmol. Astropart. Phys. 1, 20.

[ref36] Macintosh B., Graham J., Palmer D., Doyon R., Gavel D., Larkin J., Oppenheimer B., Saddlemyer L., Wallace J.K., Bauman B. (2007). Adaptive optics for direct detection of extrasolar planets: the Gemini Planet Finder. C. R. Phys. 8, 365–373.

[ref37] Malanima P. (2011). Energy consumption and energy crisis in Roman world In The Ancient Mediterranean Environment between Science and History, ed. Harris W., Columbia Studies in the Classical Tradition, Brill, Columbia, 39, 13–36.

[ref38] Maruyama K., Nori F. & Vedral V. (2009). The physics of Maxwell's demon and information. Rev. Mod. Phys. 81, 1–23.

[ref39] Michaud M.A.G. (2007). Contact with Alien Civilizations. Springer Science, NY.

[ref40] Moretto G. & Kuhn J.R. (2014). Highly sensitive telescope designs for higher contrast observations. Adv. Opt. Technol. 3(3), 251–264.

[ref41] Moretto G., Kuhn J.R., Thiebaut E., Langlois M., Berdyugina S.V., Harlingten C. & Halliday D. (2014). New strategies for an extremely large telescope dedicated to extremely high contrast: the Colossus project. Proc. SPIE, Ground-based and Airborne Telescopes V, 9145, 91451L9.

[ref42] NASA (2014). NEO program data. http://neo.sci.gsfc.nasa.gov

[ref43] Nealson K.H. & Conrad P.G. (1999). Life: past, present and future. Philos. Trans. R. Soc. Lond. B 354, 1923–1938. 10670014 10.1098/rstb.1999.0532 PMC1692713

[ref44] O'Neill G.K. (2000). The High Frontier: Human Colonies in Space: Apogee Books Space Series 12, 3rd edn Collector's Guide Publishing, Inc., Ontario, Canada, 184 p.

[ref45] Pepe F., Lovis Ch., Segransan D., Benz W., Bouchy F., Dumusque X., Mayor M., Queloz D., Santos N. & Udry S. (2011). The HARPS search for Earth-like planets in the habitable zone: I – very low-mass planets around HD20794, HD85512 and HD192310. Astron. Astrophys. 534, A58.

[ref46] Phoenix Project (2002). http://www.seti.org/seti-institute/project/details/project-phoenix

[ref47] Rizwan A.M., Leung D. & Chunho L. (2008). A review on the generation, determination and mitigation of Urban Heat Island. J. Environ. Sci. 20, 120–128. 10.1016/s1001-0742(08)60019-4 18572534

[ref48] Roelfsema R., Gisler D., Pragt J., Schmid H.M., Bazzon A., Dominik C., Baruffolo A., Beuzit J.-L., Charton J., Dohlen K. (2011). The ZIMPOL high contrast imaging polarimeter for SPHERE: subsystem test results. In *Techniques and Instrumentation for Detection of Exoplanets V*, edited by S. Shaklan, Proc. SPIE 8151: 81510N–81510N-13.

[ref49] Schneider J., Léger A., Fridlund M., White G.J., Eiroa C., Henning T., Herbst T., Lammer H., Liseau R., Paresce F., (2010). The far future of exoplanet direct characterization. Astrobiology 10, 121–126. 20307188 10.1089/ast.2009.0371

[ref50] Shklovskii I.S. & Sagan C. (1966). Intelligent Life in the Universe. Dell, NY.

[ref51] SIMBAD: Astronomical Database. http://simbad.u-strasbg.fr/simbad/

[ref53] TMT: Thirty Meter Telescope. http://www.tmt.org/

[ref54] Tuomi M., Anglada-Escudé G., Gerlach E., Jones H.R.A., Reiners A., Rivera E.J., Vogt S.S. & Butler R.P. (2012). Habitable zone super-Earth candidate in a six-planet system around the K2.5V star HD 40307. Astron. Astrophys. 549, A48.

[ref55] Wittenmyer R.A., Tuomi M., Butler R.P. (2014). GJ 832c: a super-Earth in the habitable zone. Astrophys. J. 791, 114 (11 pp).

